# Endocapillary hypercellularity levels are associated with early complete remission in children with class IV lupus nephritis as the initial presentation of SLE

**DOI:** 10.1186/s12882-022-02921-5

**Published:** 2022-08-26

**Authors:** Chunzhen Li, Yanan Han, Lili Zhang, Zhiguo Chen, Mei Jin, Suzhen Sun

**Affiliations:** 1 Department of Pediatrics, Hebei Medical University, Shijiazhuang, China; 2 Department of Pediatric Nephrology and Immunology, Children’s Hospital of Hebei Province, Shijiazhuang, China; 3 Department of Pathology, Children’s Hospital of Hebei Province, Shijiazhuang, China; 4 Department of Pediatric Thoracic Surgery, Children’s Hospital of Hebei Province, Shijiazhuang, China; 5 Department of Pediatric Neurology, Children’s Hospital of Hebei Province, 133 Jianhua South Street, Shijiazhuang, 050031 Hebei Province China

**Keywords:** Systemic lupus erythematosus, Pediatric lupus nephritis, Endocapillary hypercellularity, Complete remission

## Abstract

**Background:**

Endocapillary hypercellularity (ECHC) is commonly seen in class IV lupus nephritis (LN), the most common and severe LN in children. Factors influencing early complete remission (CR) in pediatric class IV LN have been poorly described. We investigated the relationship between ECHC levels and early CR in pediatric class IV LN.

**Methods:**

Patients with newly, simultaneously diagnosed systemic lupus erythematosus (SLE) and class IV LN by renal biopsy from 2012 to 2021 were studied. In this retrospective study, two pathologists who were blind to clinical information reviewed all pathological data retrospectively and classified glomerular lesions according to the revised criteria of the International Society of Nephrology and the Renal Pathology Society (ISN/RPS). The demographics, baseline clinical characteristics, laboratory parameters, renal histopathological findings, treatment regimen and CR at 6 months after immunosuppressive therapy were analyzed. ECHC was categorized as: > 50% (group A), 25–50% (group B) and < 25% (group C). CR was defined as absence of clinical symptoms, 24-hour urinary protein < 0.15 g, and normal levels of serum creatinine and albumin.

**Results:**

Sixty-four patients were identified: 23, 15 and 26 in groups A, B and C, respectively. Group A had significantly higher levels of D-dimer, urine protein, and SLE disease activity index (SLEDAI) than groups B and C. Group C had a markedly higher estimated glomerular filtration rate (eGFR) than groups A and B. A substantially greater proportion of patients in group A had glomerular microthrombi and basement membrane thickening than in groups B and C. At 6 months post treatment, CR was achieved in 19 (82.6%), 5 (33.3%) and 11 (42.3%) in groups A, B and C, respectively (*p* < 0.05, group A vs groups B and C). Multiple logistic regression analysis revealed that ECHC and urine protein levels were significantly associated with CR.

**Conclusion:**

ECHC and urine protein levels may be valuable biomarkers for predicting early CR in pediatric class IV LN.

## Background

Systemic lupus erythematosus (SLE) is an autoimmune disease resulting from the dysregulation of both innate and adaptive immune responses; it affects various organs, including the skin, joints, the central nervous system and the kidneys [1–4]. The pathogenesis of SLE is complex. To date, ample evidence indicates that genetic predisposition, hormonal dysregulation and environmental triggers are responsible for the increased availability of, and the loss of immune tolerance to, self-antigens [1–4], leading to the production of autoantibodies, such as anti-dsDNA, anti-SSA (Ro), anti-SSB (La), anti-ribonucleoprotein (RNP), and anti-Smith antibodies [5–7]. These autoantibodies then bind self-antigens to form immune complexes (ICs) that, through circulation, deposit on the wall of small arteries in various organs, resulting in complement activation, inflammatory reactions, and ultimately organ damage [1–4].

Lupus nephritis (LN) is a severe clinical manifestation of SLE [8], and a significant proportion of patients with new onset SLE have LN as the initial presentation [9, 10]. According to glomerular pathological features, LN is classified by the International Society of Nephrology/Renal Pathology Society (ISN/RPS) as follows: class I and II are defined as lesions involving only the mesangium; class III is focal glomerulonephritis that affects < 50% of total number of glomeruli; class IV is diffuse glomerulonephritis that involves ≥50% of total number of glomeruli; class V is membranous lupus nephritis; and class VI is characterized by advanced sclerosing lesions [11, 12].

LN is more common in childhood-onset SLE than it is in adult-onset SLE [13–15]. It occurs in 50–60% of patients with childhood-onset SLE [16]. Among the pathologically proven LN cases reported thus far, class IV is the most commonly seen LN in children [17–23]. As the most severe form of LN, class IV LN requires intensified immunosuppressive therapy [19], and currently few studies have specifically examined early treatment outcome and its influencing factors of class IV LN in children [19, 24]. As endocapillary hypercellularity (ECHC) is commonly seen in class IV LN) [11–13], and vascular injuries have been shown to affect patient prognosis in adults with LN [25–27], we hypothesized that levels of ECHC might influence early treatment outcome of pediatric class IV LN. To test this, we retrospectively analyzed baseline clinical characteristics, lab testing results, pathological findings, and the treatment outcome at 6 months post immunosuppressive therapy in children with class IV LN as the initial presentation of SLE.

## Materials and methods

This study is approved by the Medical Research Ethics Committee of Children’s Hospital of Hebei Province affiliated with Hebei Medical University (Approval #202136), in compliance with the principles of the Declaration of Helsinki, the Code of Ethics of the World Medical Association. The Ethics Committee of Children’s Hospital of Hebei Province waived the need for informed consent due to retrospective study.

### Patients

All patients with newly diagnosed SLE with class IV LN as the initial presentation determined by renal biopsy from 2012 to 2021 were included in this study. Children with acute nephritis associated with post-streptococcal disease, IgA nephropathy and Henoch-Schonlein pupura were excluded. The SLE activity was scored according to the Systemic Lupus Erythematosus Disease Activity Index 2000 (SLEDAI-2 K) [28]. The demographic data, baseline clinical characteristics, laboratory parameters, renal histopathological findings, and treatment regimen and outcome at 6 months after treatment were retrospectively reviewed and analyzed.

### Laboratory testing

Blood testing included the following parameters: white cell and platelet counts, hemoglobin (HGB), C-reactive protein (CRP), erythrocyte sedimentation rate (ESR), serum creatinine (CR), urea nitrogen, albumin, cholesterol, D-dimers, C3 and C4 complement, anti-nuclear antibodies (ANA), anti-dsDNA, anti-SSA, anti-SSB, anti-Sm, anti-RNP, anti-nucleosome (anti-NCS), anti-cardiolipin, anti-P protein and anti-histone antibodies. The estimated glomerular filtration rate (eGFR) was calculated as described elsewhere [29]. Urinalysis included white cell and red cell counts, and measurement of protein in 24 hour urine collection (mg/kg^/^day).

### Histopathological analysis

Periodic Acid-Schiff staining (PAS), Periodic Acid Schiff Methenamine Silver Staining (PASM), Masson staining, HE staining, immunofluorescence staining of IgM, IgG, IgA, C1q and C3 were carried out as per the protocols established at our pathology department. At least 10 glomeruli from each biopsy were evaluated. Two pathologists who were blind to clinical information reviewed all pathological data retrospectively, and classification of glomerular lesions was done according to the International Society of Nephrology and the Renal Pathology Society (ISN/RPS) criteria revised in 2018 [12]. The ECHC level in each biopsy was categorized as: > 50% (group A), 25–50% (group B) and < 25% (group C) [12].

### Treatment and follow-up

For induction therapy, intravenous methylprednisolone (15–20 mg/kg/day) was administered for three consecutive days (maximum dose 1 g/day) followed by intravenous cyclophosphamide (CYC) 8–12 mg/kg/day for 2 consecutive days/week, every other week for 8 times. Maintenance therapy included oral mycophenolate mofetil (MMF) at 20 mg/kg/day (maximum dose 2 g/day) if 24-hour urine protein < 0.5 g or tacrolimus at 0.05–0.1 mg/kg/day (maximum dose 4 mg/day) if 24-hour urine protein > 0.5 g.

Follow-up was conducted once every month at the outpatient department. Complete remission (CR) was defined as: absence of clinical symptoms, normal levels of serum albumin (39–54 g/L for children aged 6 to < 13 years, and 42–56 g/L for children aged 13–18 years) and creatinine (27–66 μmol/L for children aged 6 to < 13 years, 37–93 μmol/L and 33–75 μmol/L for boys and girls aged 13–16 years, respectively) (http://www.nhc.gov.cn/fzs/s7852d/202105/3f216b22ed084069b65399f969035358/files/8d4b58cf376d4b129907c19d147b8433.pdf), and 24-hour urinary protein < 0.15 g [20].

### Statistical analysis

The normality of continuous variables was assessed with the Shapiro-Wilk test. Parametric data were presented as mean ± standard deviation and analyzed with the one-way ANOVA with post hoc Tukey’s test. Non-parametric data were presented as median (first quartile, third quartile) and analyzed with the Kruskal-Wallis ANOVA with post hoc Dunn’s test. Categorical data were analyzed with the chi-square or the fisher’s test. Multiple logistic regression test was performed to determine the factors influencing the treatment outcome. All statistical analyses were conducted using the SPSS 25.0 software, and *p* < 0.05 was considered statistically significant.

## Results

A total of 64 children (47 girls) with newly, simultaneously diagnosed SLE and class IV LN by renal biopsy were treated in our hospital during 2012 to 2021. Of these patients, 23 had ECHC > 50% (group A), 15 had ECHC between 25 and 50% (group B) and 26 had ECHC < 25% (group C). The demographics and baseline clinical characteristics are shown in Table 1. There were no significant differences in age, gender, the frequency of pulmonary, cardiac, ophthalmologic and neurological involvement, or the proportion of children with hypertension among the 3 groups. No significant difference was found in the time from the first symptom onset to final diagnosis between groups A and B or groups B and C. Group A had a significantly higher SLEDAI score than groups B and C (Table 1). Rash, swelling and fever were the most common non-renal manifestations, and their frequencies in the 3 groups were not substantially different (Table 1).

**Table 1:** Demographic data and baseline clinical characteristics of all patients at admission

	**Group A (*n*** **= 23)**	**Group B (*n*** **= 15)**	**Group C (*n*** **= 26)**
Female, n	16	12	19
Age (year)	10.00 ± 2.04	10.47 ± 2.33	11.43 ± 2.50
Time from first symptoms to final diagnosis (day)	9 (5, 14) ^**△**^	15 (10, 20)	15 (10, 60) ^**△**^
Family history of SLE, n	1	1	1
Hypertension, n	14	6	15
Pulmonary involvement, n	14	10	16
Cardiac involvement, n	11	6	7
Ophthalmologic involvement, n	3	2	1
Neurological involvement, n	7	5	6
SLEDAI-2 K	28.70 ± 7.17*^△^	21.07 ± 5.18*	20.62 ± 5.67^**△**^
Rash, n	18	8	15
Swelling, n	13	8	12
Fever, n	11	7	11

Data are presented as mean ± SD or median (first quantile, third quantile)

*SLE* Systemic lupus erythematosus, *SLEDAI-2 K* The systemic lupus erythematosus disease activity index 2000

*p < 0.05 (group A compared with group B); and ^**△**^ *p* < 0.05 (group A compared with group C)

Laboratory results at admission are shown in Table 2. Group A had a significantly higher level of D-dimer, urine protein, urine WBC and urine RBC than groups B and C. Group C had a significantly higher level of eGFR and C3 than groups A and B. The positive rate of anti-SSA antibodies is markedly higher in group C than in groups A and B.

**Table 2:** Baseline laboratory parameters before treatment

Parameter	Group A (*n* = 23)	Group B (*n* = 15)	Group C (*n* = 26)
*Blood testing*			
WBC (10^9^/L)	4.80 (3.20, 7.00)	5.20 (2.90, 7.90)	5.45 (3.65, 7.45)
PLT (10^9^/L)	137.00 (96.00, 216.00)	145.00 (85.00, 206.00)	149.00 (117.50, 198.75)
ESR (mm/h)	41.23 ± 22.85	60.60 ± 28.82	52.72 ± 22.45
HGB (g/L)	90.65 ± 16.51	97.53 ± 19.62	95.69 ± 15.32
ALB (g/L)	22.77 ± 7.25	27.01 ± 8.38	28.78 ± 6.15
CHOL (mmol/L)	4.50 (3.62, 7.16)	4.33 (3.40, 6.14)	5.33 (3.86, 6.38)
CRP (mg/L)	1.00 (0.41, 2.00)	0.83 (0.50, 1.84)	0.90 (0.43, 4.40)
D-Dimer (mg/L)	3.22 (1.60, 13.60)*^△^	1.22 (0.77, 2.59)*	0.84 (0.39, 1.35)^**△**^
BUN (mmol/L)	7.32 (5.13, 11.54)	7.41 (4.23, 15.30)	7.60 (5.99, 11.78)
Serum creatinine (μmol/L)	78.00 (46.00, 102.00)	58.00 (37.00, 119.00)	62.00 (49.00, 73.50)
eGFP (ml/min/1.73m^2^)	80.77 ± 35.13*^△^	87.56 ± 55.91*	113.73 ± 44.30^**△**^
C3 (g/L)	0.22 (0.11, 0.26)^**△**^	0.19 (0.14, 0.25)^⋕^	0.39 (0.20, 0.61)^⋕**△**^
C4 (g/L)	0.02 (0.02, 0.04)*^△^	0.04 (0.03, 0.07)*	0.06 (0.03, 0.08)^**△**^
Anti-dsDNA	22 (23)	13 (15)	21 (26)
Anti-Smith	3 (23)	3 (15)	7 (26)
Anti-RNP	6 (23)	5 (15)	6 (26)
Anti-P protein	10 (23)	3 (15)	7 (26)
Anti-NCS	14 (23)	9 (15)	9 (26)
Anti-histone	8 (23)	2 (15)	8 (26)
Anti-SSA	1 (23)^**△**^	1 (15) ^⋕^	12 (26)^**△**⋕^
Anti-SSB	0 (23)^**△**^	3 (15)	7 (26)^**△**^
Anti-cardiolipin	6 (23)	2 (15)	4 (26)
*Urine testing*			
Urine WBC/HP	29.00 (10.00, 43.00)*^△^	3.00 (0.00, 24.00)*	9.00 (3.50, 17.00)^**△**^
Urine RBC/HP	165.00 (42.00, 452.00)*^△^	26.00 (10.00, 78.00)*	38.00 (11.00, 87.00)^**△**^
Urine protein (mg/kg/day)	117.73 (65.71, 168.75)*^△^	57.25 (26.32, 144.52)*	35.73 (18.31, 78.50)^**△**^

Date are presented as mean ± standard deviation or median (first quartile, third quartile)

*WBC* White blood cell, *PLT* Platelet, *ESR* Erythrocyte sedimentation rate, *HGB* Hemoglobin, *ALB* Albumin, *CHOL* Cholesterol, *CRP* C-reactive protein, *BUN* Blood urea nitrogen, *RNP* Ribonucleoprotein, *NCS* Nucleosome, *SSA* Sjögren’s-syndrome type A antigen, *SSB* Sjögren’s syndrome type B antigen, *RBC* Red blood cell, *HP* high power field

* *p* < 0.05 (group A compared with group B); ⋕*p* < 0.05 (group B compared with group C); and **△** *p* < 0.05 (group A compared with group C)

Concurrent positive staining for IgG, IgM, IgA, C3 and C1q were observed in all cases. Apart from different levels of ECHC among the 3 groups, other pathological differences are as follows: a remarkably higher proportion of patients in group A had glomerular microthrombi and glomerular basement membrane thickening compared with groups B and C (Table 3).

**Table 3:** Major histopathological findings from the biopsy

	**Group A**	**Group B**	**Group C**
Cellular crescents	2.00 (0.00, 4.00)	2.00 (0.00, 8.00)	1.00 (0.00, 4.25)
Fibrocellular crescents	0.00 (0.00, 2.00)	0.00 (0.00, 3.00)	0.00 (0.00, 1.25)
Fibrinoid necrosis	8 (23)	7 (15)	12 (26)
Leukocyte infiltration	15 (23)	6 (15)	15 (26)
Glomerular microthrombi	12 (23)*^△^	2 (15)*	6 (26)^**△**^
GBM thickening	10 (23)*^△^	0 (15)*	2 (26)^**△**^
Segmental GS	4 (23)	4 (15)	9 (26)
Tubular atrophy	9 (23)	10 (15)	16 (26)
Interstitial fibrosis	11 (23)	7 (15)	14 (26)
Arteriole wall thickening	21 (23)	14 (15)	24 (26)
Wire-loop lesion	0 (23)^**△**^	3 (15)	6 (26)^**△**^

Data of crescents are expressed as median (first quartile, third quartile)

*GBM* Glomerular basement membrane, *GS* Glomerulosclerosis

* *p* < 0.05, group A compared with B; ^**△**^ *p* < 0.05, group A compared with group C

For induction therapy, two patients in group A and one in group C received intravenous methylprednisolone for 6 days. For the rest of patients, induction therapy was given as described in the Methods. For maintenance therapy, 13, 7 and 11 patients in groups A, B and C received MMF, respectively, and 10, 8 and 15 in groups A, B and C had tacrolimus, respectively. At 6 months post treatment, CR was achieved in 19 (19/23, 82.6%), 5 (5/15, 33.3%) and 11 (11/26, 42.3%) children in groups A, B and C, respectively (*p* < 0.05 for group A vs group B and group A vs group C). Multiple logistic regression analysis revealed that the levels of ECHC and 24-hour urine protein were significantly associated with the CR rate at 6 months of therapy (Table 4) (if collinearity is present among several variables, one was chosen for analysis, such as in the case of urine protein, urine RBC and urine WBC, urine protein was used to determine the regression coefficient).

**Table 4:** Multiple logistic regression to determine variables associated with CR

Variables	OR	95% CI	*P-*value
ECHC	0.234	0.084–0.649	0.005
D-dimer	0.943	0.859–1.036	0.223
Urine protein	0.988	0.980–0.996	0.002
SLEDAI	1.052	0.986–1.123	0.128
eGFR	1.002	0.992–1.011	0.716
C3	0.360	0.031–4.207	0.415
GBM thickening	2.154	0.804–5.769	0.127

*CR* Complete remission, *ECHC* Endocapillary hypercellularity, *eGFR* Estimated glomerular filtration rate, *GBM* Glomerular basement membrane

## Discussion

In this study, we assessed CR at 6 months after immunosuppressive therapy for pediatric class IV LN, and reported the following findings: 1) children with class IV LN had varied ECHC levels; 2) those with ECHC > 50% had a higher SLEDAI score, higher levels of D-dimer and urine protein, higher urine WBC and RBC counts, and higher levels of glomerular microthrombi and glomerular basement membrane thickening but lower eGFR and C3; 3) patients with ECHC > 50% had a significantly higher CR rate; and 4) ECHC and urine protein levels were significantly associated with CR.

We aimed to investigate CR at 6 month post treatment as early CR predicts better long-term prognosis [18, 30–33], and factors that influence early CR in children with LN are rarely documented [19]. Houssiau et al. reported that long-term renal impairment defined as a serum creatinine concentration repeatedly ≥1.4 mg/dL occurred less frequently in patients who attained CR within the first 6 months of therapy than in those who did not [30]. So et al. showed that CR at 6 months of immunosuppressive therapy was associated with a significantly lower rate of end-stage renal disease (ESRD) in adults with class III or IV LN [31]. A multi-center study in the UK demonstrated that adult LN patients who had partial remission at 6 months after treatment were more likely to flare and develop ESRD compared with those who had CR [32]. Similar findings were also described for adult class IV LN by Won et al. [33]. More recently, a study investigating long term renal survival in 53 pediatric LN patients revealed that 4 children who developed ESRD after a median follow-up of 54.5 months all had class IV LN [18]. Furthermore, children who achieved CR at 6 months after induction treatment were found to have better renal survival compared with those who did not [18].

The rate of CR at 6 months of therapy in adults has been reported to vary from region to region. So et al. reported that among 117 South Korean patients ~ 50% achieved CR [31]. Hui observed a CR rate of ~ 35% at three centers in the UK [32]. Pinto Peñaranda et al. revealed a dismal CR rate of ~ 24% in a cohort of Colombian patients [34]. Similarly, different early CR rates in children have been described. A study in Singapore revealed that 43.8% (7/16) children with class IV LN achieved CR at 6 months [35]. Suhlrie et al. treated 79 predominantly Caucasian children with the vast majority (86%) having class IV LN, and assessed 12-month renal outcome. They reported a CR rate of 38% [19]. A CR rate of 23% at 12 months in a cohort of predominantly African-American children was described by Lau et al. [22]. We found a CR rate of 55% (35/64) in the present study. These discrepancies may be due to genetic disparities and different clinical statuses.

Recently, Almaani et al. revealed that glomerular mRNA abundance of several genes, *SPP1* (secreted phosphoprotein 1, also known as osteopontin) in particular, were positively associated with ECHC in adult patients with LN [36]. Furthermore, glomerular *SPP1* mRNA levels significantly decreased in patients achieving CR [36]. SPP1 is a pro-inflammatory molecule and plays an essential role in autoimmune disease by regulating Th17 cells [37]. It also activates macrophages [38], leading to the production of growth factors that are directly linked to cellular proliferation in LN [39]. These findings may be related to the phenomenon seen in our study, i.e., children with higher levels of ECHC responded better to immunosuppressive therapy. The association between dysregulated genes and ECHC [36], and our finding that patients with higher levels of ECHC responded better to treatment suggest that the dysregulated genes may be potential therapeutic targets for proliferative LN.

Virtually equal efficacy has been shown for intravenous CYC or MMF for induction therapy for severe pediatric LN [19, 20, 22]. As MMF is more expensive than CYC, CYC was used in all our cases. In addition to induction therapy, intravenous methylprednisolone pulses have been used to treat pediatric class IV LN [19, 20]. In the present study, all patients received intravenous methylprednisolone pulses.

Early CR is crucial to the prevention of long-term kidney damage caused by LN. However, features predictive of early CR for pediatric class IV LN have been rarely addressed [19]. In this study, we found that patients with different levels of ECHC had significantly different laboratory and clinicopathological parameters, some of which were correlated such as urine protein levels, and urine WBC and RBC counts. For correlated variables, only one was selected for multiple logistic regression analysis, which showed that ECHC and urine protein levels are significantly associated with CR at 6 months. Of note, a recent study identified proteinuria at the time of diagnosis as the only risk factor for not attaining CR at 12 months in children mostly with class IV LN [19], supporting our findings. Limitations of this study include: 1) retrospectively corrected date; 2) a single center study; 3) a small sample size; and 4) limited ethnic/racial diversity. Additionally, all children included in this study were newly, simultaneously diagnosed with SLE and class IV LN with a short period of illness, making it hard to analyze the effect of chronic pathological changes such as glomerular sclerosis, tubular atrophy and interstitial fibrosis on CR.

## Conclusion

In conclusion, children with class IV LN had varied degrees of ECHC. ECHC and urine protein levels are associated with early CR, and may be valuable biomarkers for predicting early CR in pediatric class IV LN.

## Data Availability

Data are available upon reasonable request made to the corresponding author.
